# MicroRNA-605-3p Inhibited the Growth and Chemoresistance of Osteosarcoma Cells *via* Negatively Modulating RAF1

**DOI:** 10.2174/0109298665314658240712051206

**Published:** 2024-07-29

**Authors:** Mao Wang, Weina Li, Guohui Han, Xiangdong Bai, Jun Xie

**Affiliations:** 1 Department of Bone and Soft Tissue Oncology, Shanxi Province Cancer Hospital/ Shanxi Hospital Affiliated to Cancer Hospital, Chinese Academy of Medical Sciences/Cancer Hospital Affiliated to Shanxi Medical University, Taiyuan, Shanxi, China;; 2 Department of Radiotherapy, Shanxi Province Cancer Hospital/ Shanxi Hospital Affiliated to Cancer Hospital, Chinese Academy of Medical Sciences/Cancer Hospital Affiliated to Shanxi Medical University, Taiyuan, Shanxi, China;; 3 Department of Breast Surgery, Shanxi Province Cancer Hospital/ Shanxi Hospital Affiliated to Cancer Hospital, Chinese Academy of Medical Sciences/Cancer Hospital Affiliated to Shanxi Medical University, Taiyuan, Shanxi, China;; 4 Department of Biochemistry and Molecular Biology, Shanxi Medical University, Taiyuan, 030001, China

**Keywords:** miR-605-3p, RAF1, osteosarcoma, ERK, MEK, OS cells

## Abstract

**Background:**

Osteosarcoma (OS) is the leading cancer-associated mortality in childhood and adolescence. Increasing evidence has demonstrated the key function of microRNAs (miRNAs) in OS development and chemoresistance. Among them, miRNA-605-3p acted as an important tumor suppressor and was frequently down-regulated in multiple cancers. However, the function of miR-650-3p in OS has not been reported.

**Objective:**

The aim of this work is to explore the novel role of miR-605-3p in osteosarcoma and its possible involvement in OS chemotherapy resistance.

**Methods:**

The expression levels of miR-605-3p in OS tissues and cells were assessed by reverse transcription quantitative PCR (RT-qPCR). The relevance of miR-605-3p with the prognosis of OS patients was determined by the Kaplan-Meier analysis. Additionally, the influence of miR-605-3p on OS cell growth was analyzed using the cell counting kit-8, colony formation assay, and flow cytometry. The mRNA and protein expression of RAF1 were detected by RT-qPCR and western blot. The binding of miR-605-3p with the 3’-UTR of RAF1 was confirmed by dual-luciferase reporter assay.

**Results:**

Our results showed that miR-605-3p was markedly decreased in OS tissues and cells. A lower level of miR-605-3p was strongly correlated with lymph node metastasis and poor 5-year overall survival rate of OS patients. *In vitro* assay found that miR-605-3p suppressed OS cell proliferation and promoted cell apoptosis. Mechanistically, the proto-oncogene RAF1 was seen as a target of miR-605-3p and strongly suppressed by miR-605-3p in OS cells. Restoration of RAF1 markedly eliminated the inhibitory effect of miR-605-3p on OS progression, suggesting RAF1 as a key mediator of miR-605-3p. Consistent with the decreased level of RAF1, miR-605-3p suppressed the activation of both MEK and ERK in OS cells, which are the targets of RAF1. Moreover, lower levels of miR-605-3p were found in chemoresistant OS patients, and down-regulated miR-605-3p increased the resistance of OS cells to therapeutic agents.

**Conclusion:**

Our data revealed that miR-605-3p serves as a tumor suppressor gene by regulating RAF1 and increasing the chemosensitivity of OS cells, which provided the novel working mechanism of miR-605-3p in OS. Engineering stable nanovesicles that could efficiently deliver miR-605-3p with therapeutic activity into tumors could be a promising therapeutic approach for the treatment of OS.

## 1 INTRODUCTION

Osteosarcoma (OS), one of the most frequent malignancies, frequently occurs in childhood and adolescence [1, 2]. Current treatments, including surgery, chemo- and radio-therapy, have improved the outcome of OS patients [2, 3]. Nevertheless, the prognosis of advanced patients remains unsatisfactory. Therefore, exploring novel mechanisms underlying the malignancy of OS is urgent in developing an efficient treatment approach.

MicroRNAs (miRNAs), a group of small RNA molecules, are ~22 nucleotides in length without protein-coding capacity [4]. MiRNAs serve as negative gene expression regulators *via* interacting with the 3’-untranslated region (UTR) of target mRNAs [5, 6], thus leading to mRNA degradation or protein translation repression [6]. Due to the critical function of miRNAs in regulating gene expression, miRNAs play a key role in the growth, differentiation, and apoptosis of cells. Dysfunction of miRNAs has been found in human cancers, which could either promote or suppress cancer progression depending on the cellular context [7-9]. In OS, there are an increasing number of miRNAs that are deregulated and modulate the malignancy or drug resistance *via* repressing the expression of targets [3, 10-14]. These discoveries suggested the promising diagnostic and therapeutic significance of miRNAs in OS. Recent studies demonstrated that miR-605-3p acted as a tumor suppressor gene, has always shown a decreased expression level in tumor tissues [15-17]. Overexpression of miR-605-3p suppressed the growth, invasion, and migration of prostate cancer cells [15]. Lower expression of miR-605-3p was also reported in gastric cancer [18]. The tumor suppressive role of miR-605-3p was also demonstrated by a recent report that miR-605-3p, which was down-regulated in glioma, led to reduced proliferation, migration, and invasion of glioma cells [19]. Collectively, these studies supported that miR-605-3p could serve as a tumor suppressor gene in many cancers. However, to our knowledge, the involvement of miR-605-3p and novel working mechanisms in OS are still unclear.

Increasing evidence suggests that miRNA-based therapeutics, either restoring or inhibiting the function of miRNA, show potential and promising applications in cancer treatment [20-22]. However, the efficient delivery of miRNAs into target tissues is a major challenge in translating miRNA therapy into clinical practice. Considering the shortcomings of conventional medicines, nanomedicine-based tumor therapeutics show attractive promising in improving tumor targeting and therapeutic effectiveness [20-22]. These excellent studies reported the advantages and perspective of nanomedicine in cancer therapy, as well as issues that remain to be resolved. Nanocarriers such as liposome, exosome, and polymer-mediated miRNA delivery are necessary to enable the stability and location of miRNA. Considering the down-regulation of miR-605-3p in cancers, miR-605-3p carried by nanomaterials might be an alternative therapeutic strategy.

In our project, a lower level of miR-605-3p was found to be down-regulated in OS tumor samples and cell lines. miR-605-3p suppressed the growth and promoted apoptosis of OS cells. Moreover, functional studies demonstrated RAF1, also named as CRAF or proto-oncogene c-RAF, as the target of miR-605-3p. Restoration of RAF1 partially alleviated the suppressive role of miR-605-3p during OS progression.

## 2 MATERIALS AND METHODS

### 2.1 Clinical Specimens

Fifty paired tumor tissues and adjacent normal tissues were collected from OS patients who had undergone surgery at Shanxi Province Cancer Hospital. No patients received chemotherapy or radiotherapy before tissue collection. Samples were immediately stored at -80ºC until the RNA extraction. Written informed consents were obtained from all the patients. All the clinical data of the involved patients were obtained from the Department of Bone and Soft Tissue Oncology, Shanxi Province Cancer Hospital. This research has been approved by the authors’ affiliated institutions.

### 2.2 Cell Culture

Human OS cell lines U2OS, MG-63, Saos-2, HOS, and normal osteoblasts hFOB1.19 were obtained from American Type Culture Collection (ATCC), were cultured in DMEM or McCoy’s 5A medium (Gibco) with 10% fetal bovine serum (FBS, Gibco) and 1% penicillin/streptomycin (Cat#C0222; Beyotime) in an incubator at 37ºC with 5% CO_2_. Cisplatin, Sorafenib, and Doxorubicin (Dox) were purchased from MedChem Express (Shanghai, China). The control miRNA, miR-605-3p mimics and antagomir were provided by GenePharma (Shanghai, China). Cell transfection was conducted using lipofectamine 3000 according to the manufacturer’s protocol.

### 2.3 RNA Extraction and Real-time qPCR

Total RNA was extracted from tissues or cells with the TRIzol reagent (Cat# 15596018; Thermo Fisher Scientific). The RNA concentration was measured with the NanoDrop^TM^2000. cDNA was synthesized with a TaqMan MicroRNA Reverse Transcription Kit (Cat# 4366596; Thermo Fisher Scientific) according to the manufacturer’s protocol. miR-605-3p expression was evaluated using the TaqMan MicroRNA Assay Kit (Takara, Dalian, China) on the LightCycler 480 real-time PCR system. U6 RNA was detected as the internal control. The thermocycling conditions for qPCR were 95ºC for 30 s, followed by 40 cycles of 95ºC for 10s and 60ºC for 10 s. The relative gene expression was measured with the 2^-ΔΔCT^ method. The primers used in this study are listed in Table **S1**.

### 2.4 CCK-8 Cell Counting Assay

The growth of OS cells was measured with the CCK-8 Cell Counting Kit (Cat#A311-01; Vazyme) according to the manufacturer’s instructions. In general, a density of 2,000 cells, which were transfected with NC or miR-605-3p, were seeded into a 96-well plate, followed by adding 10 μl CCK-8 solution into each well for the indicated time at 37ºC. The OD value was measured at 450 nm with a microplate reader.

### 2.5 Cell Apoptosis

Cell transfection was performed to overexpress miR-605-3p, followed by detecting cell apoptosis using the Annexin V-FITC/PI Apoptosis Detection Kit (Cat# BMS500FI-20; Thermo Fisher Scientific). In brief, cells were harvested and washed by ice-cold PBS twice and then fixed with alcohol. After centrifugation, cells were suspended using the incubation buffer, followed by the addition of 5 μl Annexin V-FITC and 5 μl propidium iodide (PI). Cells were incubated in the dark for 15 min. The apoptotic cells were detected by flow cytometry (FACS Calibur; BD Sciences) and analyzed with the FlowJo software.

### 2.6 Western Blotting

Total proteins from OS cells were prepared using the NP-40 lysis buffer (Cat#FNN0021; Thermo Fisher Scientific) with the protease inhibitor cocktails (Cat# S8820; Sigma). The protein concentration was detected by the Bradford protein assay kit (Cat#5000001; Bio-Rad). The protein lysates were mixed with 5x protein loading buffer (Cat#P0015; beyotime), followed by incubating in a 100ºC heat block for 10 mins. Then, 15% SDS-PAGE gel was employed to separate proteins, followed by transferring them onto the PVDF membranes at 55 v for 120 minutes by the wet transfer method. Next, the membranes were firstly blocked with 5% non-fat milk, followed by incubating with primary antibodies overnight at 4ºC. The membranes were incubated with the secondary antibodies for 1 h at room temperature (RT). The signals were developed with the enhanced chemiluminescence substrate (Cat#32109, Thermo Fisher Scientific). Antibodies against RAF1 (Ab230850; Abcam; USA) and GAPDH (G8795; ProteinTech Group, Chicago, IL, USA), MEK1 (#2352, Cell Signaling Technology (CST), Beverly, MA, USA), p-MEK1 (S298, #9128, CST, Beverly, MA, USA), ERK1/2 (#4695, CST, Beverly, MA, USA), and p-ERK1/2 (T202/Y204; #4370, CST, Beverly, MA, USA) were commercially obtained. The blots were captured using the Tanon 5200 Chemiluminescent Imaging System (Tanon; China) according to the guidelines.

### 2.7 miR-605-3p Target Prediction and Luciferase Reporter Assay Validation

The putative binding targets of miR-605-3p were predicted by the miRDB website (https://mirdb.org). The 3’-UTR sequence of wild-type or mutated RAF1 containing the predicted binding site of miR-605-3p was synthesized by GenePharma (Shanghai, China) and constructed into the p-MIR luciferase vector. OS cells seeded in the 96-well plate were transfected with wild-type or mutated luciferase reporter along with miR-605-3p mimics or miR-NC for 48 h. The luciferase activity was measured using the Dual-Luciferase reporter assay kit (Promega) and shown as the ratios by normalizing the *firefly* activity to that of *renilla*.

### 2.8 Cell Migration Assay

OS cells were plated in 6-well plates and transfected with miR-605-3p mimics or miR-NC. After being cultured overnight, scratches were generated using the pipette tip, and the wound healing was observed after 24 hours.

### 2.9 Chemosensitivity Assay

OS cells were seeded into the 96-well plate and treated with different concentrations (0, 1, 2, 5, 10, 20, 50, and 100 μg/ml) of cisplatin or dox for 48 h. The IC_50_ values were calculated by the concentration of cisplatin or Dox that reduced the cell viability by 50% at 48 h, respectively.

### 2.10 Statistical Analysis

All data were shown as mean ± SD. The statistical analysis was calculated using GraphPad Prism 7.0 (GraphPad Software, Inc., the USA). Significant differences between different groups were calculated using the Student’s *t*-test or one-way analysis of variance followed by Tukey’s analysis. The correlation was determined by the Spearman correlation test. A *P* value less than 0.05 was determined as a statistical significance.

## 3 RESULTS

### 3.1 miR-605-3p was Down-regulated in OS and Correlated with the Poor Outcomes of OS Patients

**Figure 1:**
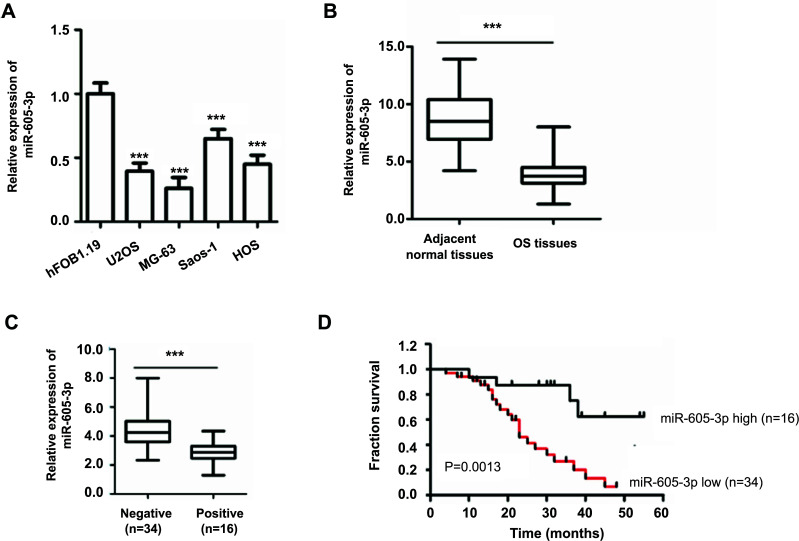
A considerable decrease of miR-605-3p was found in OS. (**A**) Abundance of miR-605-3p in OS cell lines and normal cells was detected. (**B**) Abundance of miR-605-3p in OS tumor samples and paired normal tissues was detected by RT-qPCR. Student’s *t* test was employed to calculate the *p* value. ***P<0.001 (**C**) A low level of miR-605-3p was detected in OS patient with lymph node metastasis. (**D**) Log rank test showed that patients carrying low level of miR-605-3p had relative shorter 5-year overall survival rate.

To illustrate the function of miR-605-3p during OS progression, miR-605-3p expression in OS cell lines and normal cells was detected by RT-qPCR. Our data revealed that a remarkably decreased expression of miR-605-3p was found in OS cells in comparison with that of normal hFOB1.19 cells (Figure **1A**). To confirm further the aberrant expression of miR-605-3p in OS progression, the abundance of miR-605-3p in paired OS tumor tissue and paired normal tissues was examined. Our data demonstrated that miR-605-3p was markedly reduced in OS tissues than that of paired non-cancer tissues (Figure **1B**). Additionally, as shown in Figure (**1C**), patients with lymph node metastasis displayed a relatively lower level of miR-605-3p compared with those without lymph node metastasis. Taken together, our studies documented a reduced expression of miR-605-3p during OS progression.

To further detect the correlation between miR-605-3p expression and the clinical outcome of OS patients, those patients were divided into a higher expression group or a lower expression group based on the median value of miR-605-3p. As presented in Figure (**1D**), the Kaplan-Meier curve showed that patients who carried lower levels of miR-605-3p had a shorter 5-year overall survival rate. As a result, our results documented the potential role of miR-605-3p in OS progression.

### 3.2 miR-605-3p Suppressed OS Cell Growth and Induced Cell Apoptosis

**Figure 2:**
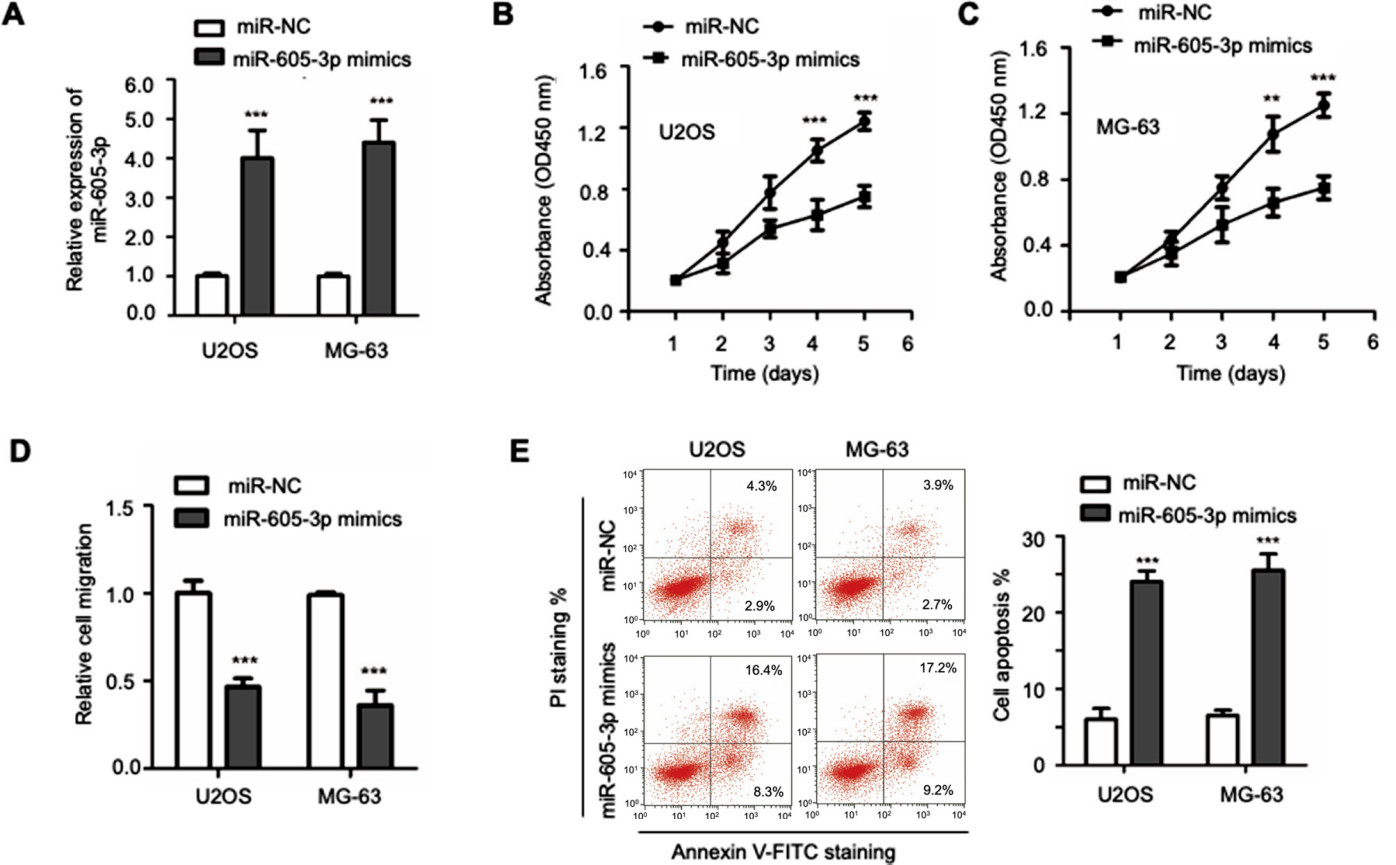
Ectopically expressed miR-605-3p inhibited the growth of OS cells. (**A**) RT-qPCR was employed to measure the overexpression efficiency of miR-605-3p. CCK-8 experiment showing the cell proliferation curves for U2OS (**B**) and MG-603 cells (**C**) expressing miR-605-3p or miR-NC. (**D**) The migration of OS cells was measured after transfected with miR-605-3p mimics. (**E**) Apoptosis cells with the transfection of miR-605-3p mimics were examined by staining with Annexin V-FITC/PI. Light panel, the representative graphs of cell apoptosis distribution. Right panel, the statistical analysis from three independent repeats. **P<0.01 and ***P<0.001.

To investigate the function of miR-605-3p in the development of OS, U2OS and MG-63 cells were transfected with miR-605-3p mimics or miR-NC, and the overexpression efficiency was measured by RT-qPCR (Figure **2A**). The CCK-8 assay was employed to determine the role of miR-605-3p on OS cell growth. Our results illustrated that overexpression of miR-605-3p resulted in a significantly decreased growth rate of these two cell lines (Figures **2B** and **2C**). In addition, transfection of miR-605-3p also obviously reduced the migration of OS cells in comparison with cells transfected with miR-NC (Figure **2D**). To further examine whether cell apoptosis was responsible for the down-regulated proliferation of OS cells, flow cytometry analysis was employed to detect the transfected U2OS and MG-63 cells. The results suggested that cells expressing miR-605-3p mimics displayed a higher percentage of apoptosis (Figure **2E**). Thus, our results indicated the suppressive role of miR-605-3p in OS cell growth.

### 3.3 RAF1 was a Target of miR-605-3p in OS

**Figure 3:**
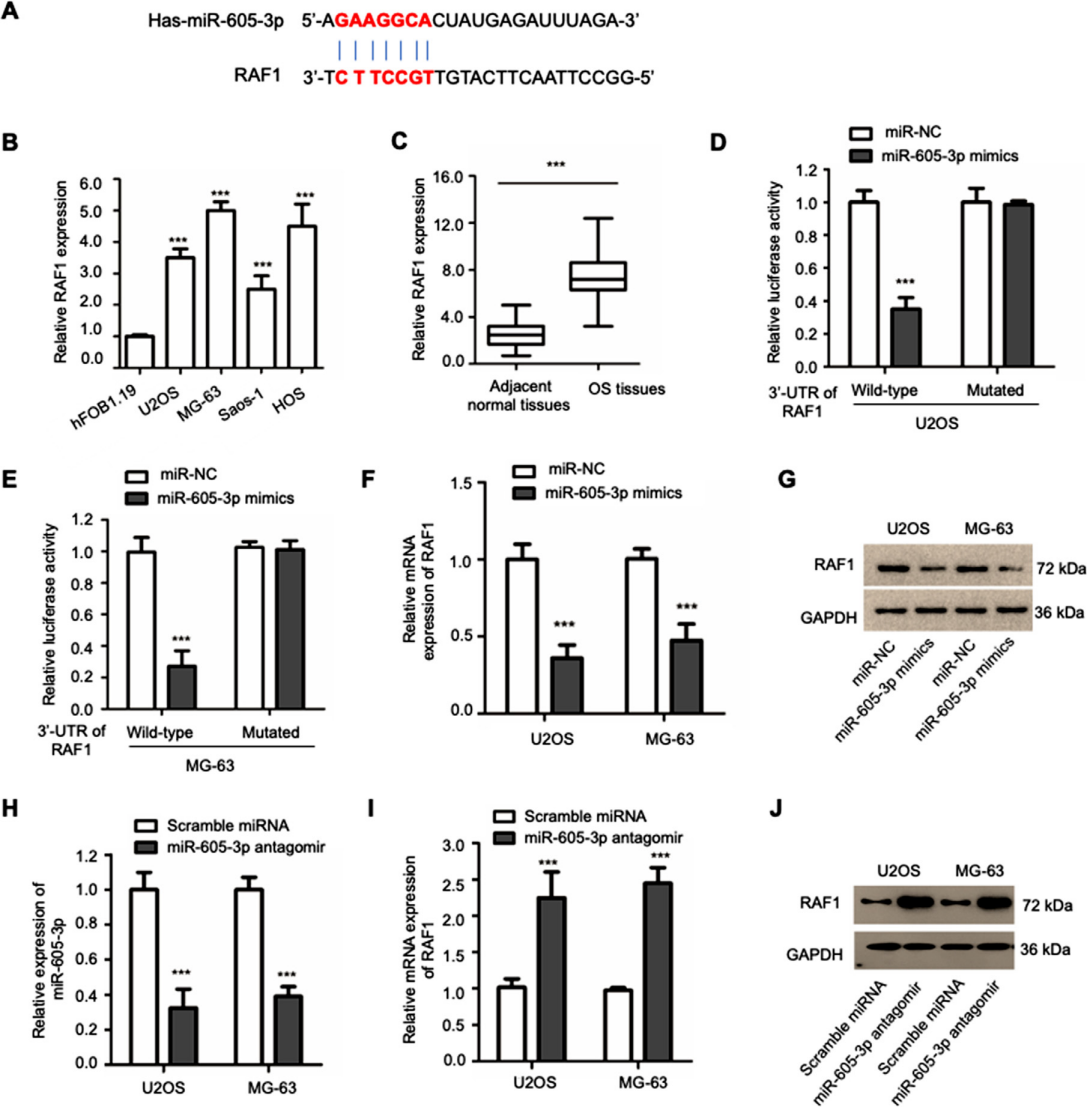
RAF1 was a target of miR-605-3p in OS. (**A**) The figure to show the predicted binding sequence of miR-605-3p at the 3′-UTR of RAF1. (**B**) The level of RAF1 in OS cells and normal hFOB1.19 cell was examined by RT-qPCR. (**C**) Abundance of miR-605-3p in OS tumor samples and paired normal tissues was detected by RT-qPCR. Student’s *t* test was employed to calculate the *p* value. ***P<0.001. (**D**, **E**) The relative luciferase activity of OS cells with WT or mutant 3’-UTR of RAF1 after transfected with miR-605-3p mimics. RT-qPCR (**F**) or western blot (**G**) was employed to detect the mRNA level and protein level of RAF1. (**H**) miR-605-3p antagomir was employed to silence miR-605-3p. The knockdown efficiency was examined by RT-qPCR. (**I, J**) miR-605-3p silencing led to an increased level of RAF1 in OS cells. ***P<0.001.

It is well documented that miRNAs regulate cancer development by modulating the expression of target genes. To further understand the function of miR-605-3p in OS, the potential targets of miR-605-3p were predicted using bioinformatics studies. From the analysis, the putative binding sites of miR-605-3p at the 3′-UTR of RAF1 are presented in Figure (**3A**). RAF1 ranked top among all the predicted targets of miR-605-3p in the online miRDB database, which deserved further analysis. Additionally, RAF1 is a pro-oncogene that displays a significant role in the development and metastasis of cancers. According to recent reports, RAF1 was overexpressed in a variety of cancers and down-regulation of RAF1 inhibited cancer progression [23-26], which means targeting RAF1 may be a promising strategy to interrupt cancer cell growth. In order to examine the potential function of RAF1 in OS, RT-qPCR was employed to detect the expression level of RAF1, which indicated an increased level of RAF1 in OS cells in comparison with that of normal hFOB1.19 cells (Figure **3B**). Moreover, consistent with previous data, a higher level of RAF1 was displayed in OS tumor tissues compared with the adjacent non-cancer tissues (Figure **3C**).

In order to further investigate the underlying mechanism, OS cells transfected with miR-605-3p and luciferase plasmids containing wild-type (WT) or mutant (MT) 3′-UTR of RAF1 were subjected to luciferase reporter assay. The results showed that miR-605-3p led to a considerable decrease in the luciferase activity of cells transfected with WT but not MUT RAF1 3’-UTR (Figures **3D** and **3E**), indicating the interaction between miR-605-3p and the 3’-UTR of RAF1. To examine whether the mRNA level of RAF1 was regulated by miR-605-3p, the mRNA expression of RAF1 in two cells transfected with miR-605-3p or miR-NC was detected by RT-qPCR. Our studies revealed that overexpression of miR-605-3p obviously suppressed the mRNA abundance of RAF1 in both OS cells (Figure **3F**). Consistently, western blot analysis also indicated that overexpression of miR-605-3p gave rise to a decreased protein level of RAF1 (Figure **3G**). In order to further validate that RAF1 was regulated by miR-605-3p, miR-605-3p antagomir or scramble miRNA was employed. The knockdown efficiency of miR-605-3p was confirmed, as indicated in Figure (**3H**). These data showed that the depletion of miR-605-3p obviously promoted the expression of RAF1 in OS cells (Figures **3I** and **3J**). Collectively, these findings identified RAF1 as a target of miR-605-3p in OS.

### 3.4 Transfection of RAF1 Rescued the Inhibitory effect of miR-605-3p on the Growth of OS Cells

**Figure 4:**
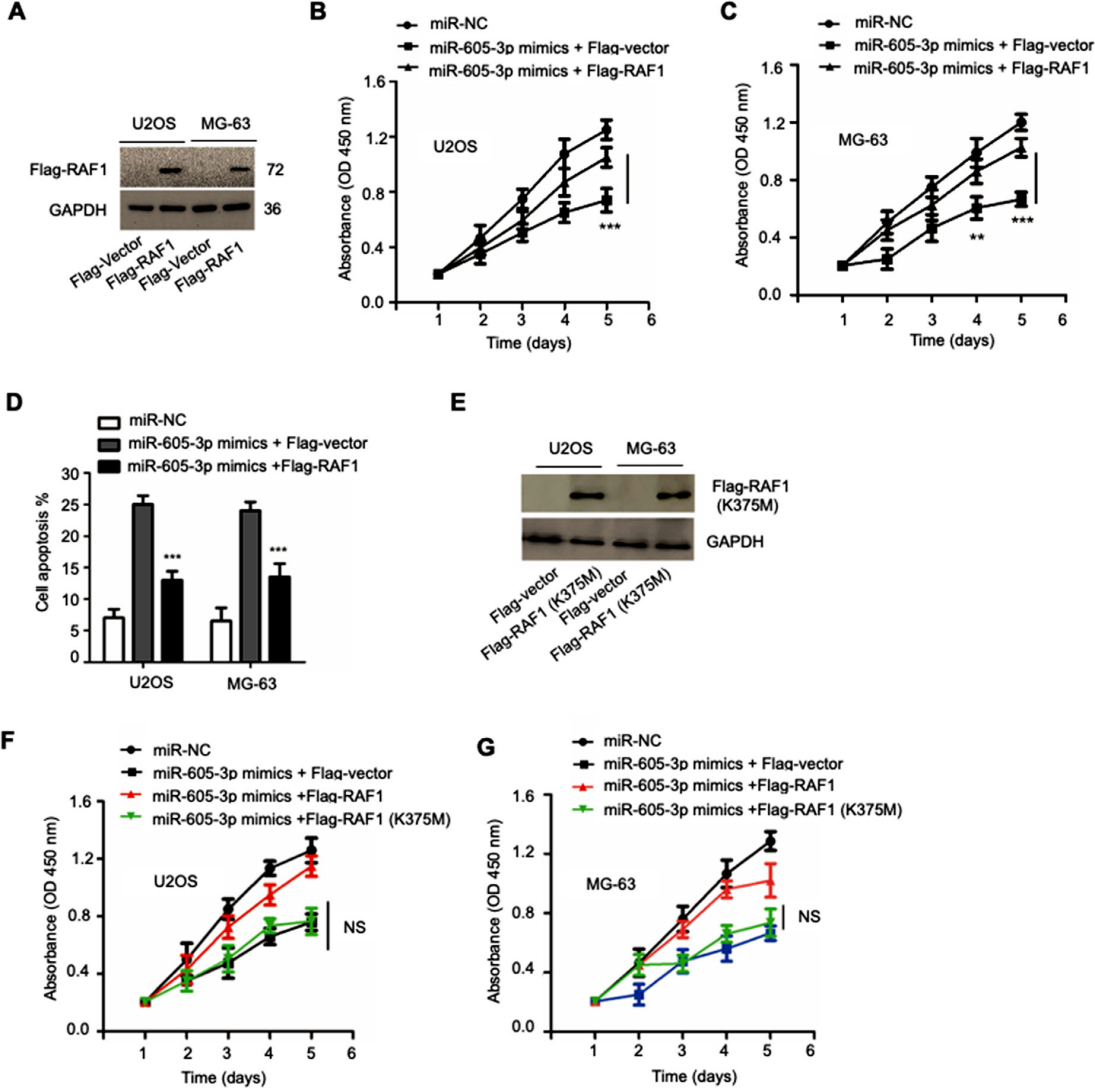
Ectopically expressed RAF1 alleviated the inhibitory effect of miR-605-3p on the growth of OS cells. (**A**) Cells were transfected with pcDNA-Flag-RAF1 or the empty vector, followed by detecting the protein level using western blot. (**B**, **C**) Ectopically expressed RAF1 alleviated the inhibitory effect of miR-605-3p on the growth of U2OS and MG-63 cells. (**D**) RAF1 transfection abrogated the pro-apoptotic effect of miR-605-3p in OS cells. (**E**) Cells were transfected with kinase-negative mutant Flag-RAF1 (K375M) and the protein expression was examined. (**F, G**) Overexpression of RAF1 (K375M) failed to rescue the growth inhibition of OS cells induced by miR-605-3p. **P<0.01 and ***P<0.001.

To further demonstrate whether the suppressive role of miR-605-3p on the malignancy of OS cells was achieved by regulating RAF1, both OS cells were transfected with pcDNA-Flag-RAF1. Western blot confirmed the up-regulation of RAF1 in OS cells after transfection for 48 h (Figure **4A**). As shown in Figures (**4B** and **4C**), compared with cells only expressing miR-605-3p, overexpression of both miR-605-3p and RAF1 obviously promoted the growth of both U2OS and MG-63 cells. Meanwhile, RAF1 overexpression also led to a lower percentage of apoptosis in OS cells in comparison with cells expressing miR-605-3p (Figure **4D**). To determine whether the kinase activity of RAF1 was involved in the inhibitory effect of miR-605-3p, the kinase-negative mutant RAF-1 (K375M) was constructed (Figure **4E**). Co-transfection of RAF1 (K375M) with miR-605-3p showed that wild-type RAF1 could rescue miR-605-3p-induced growth inhibition of OS cells, while overexpression of RAF1 (K375M) couldn’t significantly rescue the cell proliferation (Figure **4F** and **4G**). As a consequence, our data demonstrated that the tumor suppressive effect of miR-605-3p in OS was achieved mainly by RAF1.

### 3.5 miR-605-3p Inhibited RAF1 and Inactivated the MEK/ERK Pathway

**Figure 5:**
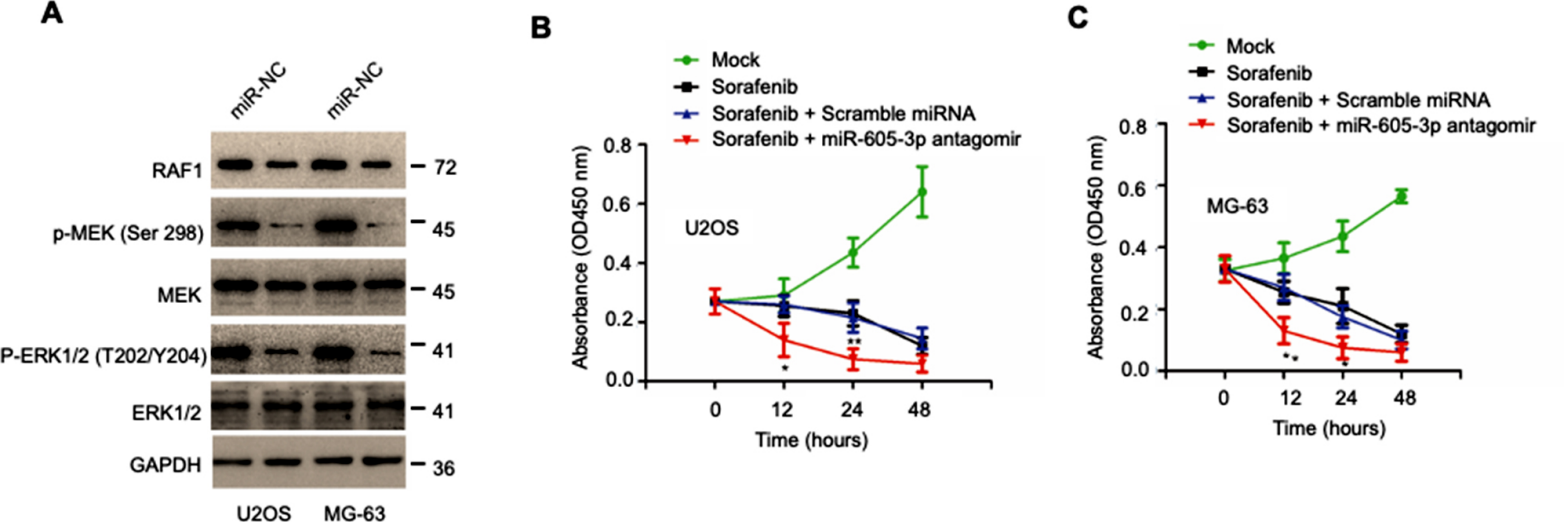
miR-605-3p inhibited the activation of MEK/ERK pathway. (**A**) The expression of RAF1, as well as the phosphorylation of MEK/ERK was measured by western blot with indicated antibodies in OS cells expressing control or miR-605-3p. (**B, C**) Cells were treated with 20 μM sorafenib for indicated hours. miR-605-3p silencing made the cells more sensitive to sorafenib treatment.

It is widely accepted that the RAF1/MEK1/ERK signaling pathway is one of the most commonly dysregulated pathways involved in cancer development. The effects of miR-605-3p on MEK1/ERK were also examined because RAF1 acted as an upstream regulator of MEK1/ERK. The phosphorylation of MEK1 at S298 and ERK1/2 at T202/Y204, which activated the function of MEK1/ERK, was examined in OS cells with overexpression of miR-605-3p. Consistent with the reduced level of RAF1, both OS cell lines over-expressing miR-605-3p revealed a repressed level of phosphorylation of MEK1 (S298), ERK1/2 (T202/Y204) (Figure **5A**). These results provided the possible mechanism to explain how reduced RAF1 by miR-605-3p affected the progression of OS.

In order to investigate the effects of increased RAF1 on U2OS and MG-603 cells, cells were treated with Sorafenib, the inhibitor of RAF1, and cell proliferation was compared. The data showed that compared with cells treated with Sorafenib alone, down-regulation of miR-605-3p made cells more sensitive to RAF1 inhibitors (Figures **5B** and **5C**). This result suggested that targeting the kinase activity of RAF1 might benefit the treatment of OS with down-regulated miR-605-3p.

### 3.6 miR-605-3p was Down-regulated in Chemo-resistant Osteosarcoma Tissues and Depletion of miR-605-3p Increased Chemoresistance of OS Cells

**Figure 6:**
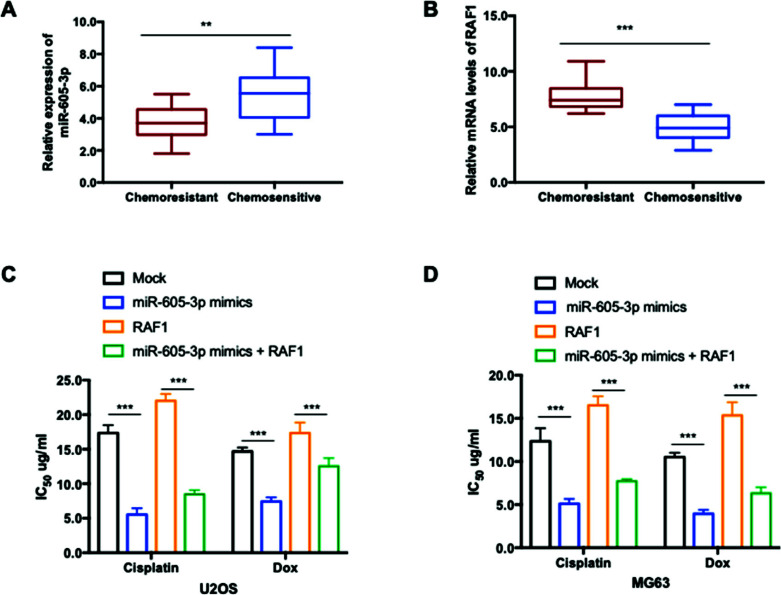
miR-605-3p was down-regulated in chemo-resistant osteosarcoma tissues and depletion of miR-605-3p increased chemoresistance of OS cells. (**A**) miR-605-3p expression in chemoresistant (n=20) and chemosensitive (n=20) OS patients was detected by RT-qPCR. Lower expression of miR-605-3p in chemoresistant OS patients was observed. (**B**) RAF1 mRNA levels were significantly increased in chemoresistant OS patients than that of those chemosensitive patients. (**C, D**) The IC_50_ values of U2OS or MG-63 cells transfected with miR-605-3p in combination with or without RAF1 after treatment with chemotherapeutic agents for 48 h. **P<0.01 and ***P<0.001.

Chemoresistance is considered a main cause of recurrence or failure of OS treatment. To determine whether aberrantly expressed miR-605-3p contributed to the chemoresistance development of OS, miR-605-3p expression was detected in tissues from 20 chemo-resistant patients and 20 cases of chemo-sensitive patients. The data showed that expression of miR-605-3p was significantly lower in OS tissues with chemoresistance, compared with those chemo-sensitive tissues (Figure **6A**). Additionally, RAF1 was also found to be up-regulated in chemo-resistant OS patients (Figure **6B**). To test the influence of miR-605-3p on the sensitivity of OS cells to chemotherapy agents, both U2OS and MG603 cells with overexpressed miR-605-3p were exposed to cisplatin or Dox. As indicated in Figures (**6C**, **D**), miR-605-3p increased the IC_50_ values of cells to the chemotherapeutic agents, while transfection of RAF1 reversed the chemotherapy sensitivity of OS cells.

## 4 DISCUSSION

Osteosarcoma ranks top among childhood and adolescence and its incidence rate has continued to rise. Moreover, it is well documented that OS is considered a faster proliferative type with a poor clinical outcome [2]. Special genetic alternations, including tumor suppressors or oncogenes induce the progression of OS. Interestingly, accumulating evidence also suggested the significant role of miRNAs in promoting OS progression, which would assist in providing novel targets for the prevention and therapy of OS [10, 13, 27]. The potential tumor suppressor miR-605-3p has shown a decreased expression level in human cancers [15, 19, 28]. The findings of the present study demonstrated a considerably decreased expression of miR-605-3p in OS tumor samples and cells. Deregulation of miR-605-3p was significantly associated with lymph node metastasis. Besides, OS patients with a higher level of miR-605-3p displayed a better 5-year overall survival. Moreover, functional studies suggested that miR-605-3p suppressed the malignant behaviors of OS, indicating that miR-605-3p could serve as a tumor suppressor gene during OS progression.

It is urgent to identify the potential targets of miRNAs to illustrate the underlying mechanism, which also provides new promising anti-cancer therapeutic targets. RAF1, a well-known oncogene in multiple cancers, was identified as a potential target of miR-605-3p in OS. RAF1 partly contributes to the MEK/ERK signal transduction and modulates many cellular processes, including cell proliferation, migration and differentiation [26]. Aberrant expression of RAF1 was found in many types of cancers and is related to tumor angiogenesis [25]. Notably, inhibition of RAF1 activity by miRNAs impaired the progression of cancers [29-31]. For example, highly expressed miR-195 inhibited the expression of RAF1 and blocked the proliferation of thyroid cancer [32]. Similarly, miR-497 achieved its anti-tumor role by negative regulation of the MAPK/ERK signaling pathway *via* targeting RAF1 in cervical cancer [33]. Previous research also revealed that miR-455 inhibited the aggressive behaviors of colorectal cancer cells by down-regulating RAF1, suggesting the potential therapeutic significance of miR-455/RAF1 signaling in the treatment of colorectal carcinoma [30]. In our project, miR-605-3p bound the 3’-UTR of RAF1 and negatively regulated RAF1 expression in OS cells. Furthermore, restoration of RAF1 obviously reversed the tumor suppressive effect of miR-605-3p in OS. Taken together, our studies revealed a vital function of miR-605-3p in OS cell growth *via* targeting RAF1. The present study provided a novel insight into the molecular mechanism by which miR-605-3p suppressed the malignant behaviors of OS. In addition to RAF1, other genes were also predicted as the potential binding targets of miR-605-3p using the miRDB online database. In this study, overexpression of RAF1 significantly rescued the function of miR-605-3p in OS, suggesting RAF1 was a key mediator of miR-605-3p. Other targets that might be involved in the function of miR-605-3p in OS also deserve more investigation. Consistent with the reduced level of RAF1, miR-605-3p also inhibited the activities of MEK/ERK signals in OS cells. These findings provided the possibility for the further use of miR-605-3p as a potential approach for OS treatment. Thus, it is an urgent need to confirm the inhibitory effect of the miR-605-3p/RAF1 axis in OS with large-scale and long-term follow-up investigation. Additionally, the tumor suppressive function of miR-605-3p in OS will be verified by *in vivo* studies.

Additionally, we proposed several mechanisms that may explain the down-regulation of miR-605-3p in OS. Recent reports have revealed almost 50% of miRNAs are located in the cancer-associated genomic regions that are lost or amplified during tumorigenesis [6]. The loss of heterozygosity for the gene locus of miR-605-3p might result in the down-regulation of miR-605-3p in OS. It was also reported that epigenetic changes can affect the expression of miRNAs [6, 10]. Aberrant DNA demethylation or histone deacetylation might contribute to the decreased transcription of miR-605-3p during OS development. Since miRNAs are processed from the pre-mature miRNA, it is a complicated process and involves many key factors [6]. Dysfunction of these regulators may lead to the reduced production of mature miR-605-3p in OS. It would be interesting to further investigate the molecular mechanisms that contribute to the down-regulation of miR-605-3p in OS.

## CONCLUSION

Our study illustrated a considerably reduced expression level of miR-605-3p in OS. The tumor suppressive effect of miR-605-3p in OS progression was mediated by RAF1, thus indicating that miR-605-3p could be a potential therapeutic approach for OS treatment.

## Data Availability

Data are available from the corresponding author upon request.

## LIST OF ABBREVIATIONS

OS: Osteosarcoma
UTR: 3’-untranslated Region
RT: Room Temperature
