# Supplementary material for: MicroRNA-605-3p Inhibited the Growth and Chemoresistance of Osteosarcoma Cells via Negatively Modulating RAF1
Source: Protein Pept Lett. 2024 Jul 29;31(7):559–68. doi: 10.2174/0109298665314658240712051206 (PMC11497142; doi:10.2174/0109298665314658240712051206)
Supplement: Supplementary file 1 [file PPL-31-559_SD1.pdf]

Supplementary Materials

Microrna-605-3p Inhibited the Growth and Chemoresistance of Osteosarcoma Cells via Negatively Modulating RAF1

Mao Wang[1], Weina Li[2], Guohui Han[3], Xiangdong Bai[3,*] and Jun Xie[4,*]

[1]Department of Bone and Soft Tissue Oncology, Shanxi Province Cancer Hospital/ Shanxi Hospital Affiliated to Cancer Hospital, Chinese Academy of Medical Sciences/Cancer Hospital Affiliated to Shanxi Medical University, Taiyuan, Shanxi, China; [2]Department of radiotherapy, Shanxi Province Cancer Hospital/ Shanxi Hospital Affiliated to Cancer Hospital, Chinese Academy of Medical Sciences/Cancer Hospital Affiliated to Shanxi Medical University, Taiyuan, Shanxi, China; [3]Department of Breast Surgery, Shanxi Province Cancer Hospital/ Shanxi Hospital Affiliated to Cancer Hospital, Chinese Academy of Medical Sciences/Cancer Hospital Affiliated to Shanxi Medical University, Taiyuan, Shanxi, China; [4]Department of Biochemistry and Molecular Biology, Shanxi Medical University, Taiyuan, 030001, China

Table S1. Primers used for the RT-qPCR assay.

Primer	Sequence (5’-3’)
miR-605-3p forward	AACGAGACGACGACAGAC
miR-605-3p reverse	AGAAGGCACTATGAGATTTAGA
U6 RNA forward	CTCGCTTCGGCAGCACA
U6 RNA reverse	AACGCTTCACGAATTTGCGT

0929-8665/24 © 2024 Bentham Science Publishers
